# Site Defects and Structural Alignment Enhance Interfacial Charge Mobility in Heterostructured Carbon Nitride Catalysts

**DOI:** 10.1021/acsnano.5c15285

**Published:** 2026-01-05

**Authors:** Teodor Jianu, Horaţiu Szalad, Vladimir Roddatis, Markus Antonietti, Nadezda V. Tarakina

**Affiliations:** † Department of Colloid Chemistry, 28321 Max Planck Institute of Colloids and Interfaces, Potsdam 14476, Germany; ‡ INM-Leibniz Institute for New Materials, Campus D2 2, Saarbrücken 66123, Germany; § Department of Materials Science and Engineering, Saarland University, Saarbrücken 66123, Germany; ∥ 28337 GFZ Helmholtz Centre for Geosciences, Telegrafenberg, Potsdam 14473, Germany

**Keywords:** organic interfaces, carbon nitrides, electron diffraction, valence EELS, ab initio simulations, oxygen reduction reaction

## Abstract

Engineering interfaces between organic semiconductors is an effective way to tailor organic electronic device performance, as charge transport and light interaction efficiency are strongly influenced by electronic coupling at molecular interfaces. Scanning transmission electron microscopy is routinely used to analyze interfaces at the atomic scale; however, its use for organic materials is limited due to the electron beam sensitivity of organic molecules, buried interfaces, and the semicrystalline nature of organics. In this work, we developed a workflow to correlate charge behavior at organic interfaces with their chemistry and structure, even when interface components are chemically and structurally similar and mixed at the nanoscale. We used this workflow to reveal the nanoscale mechanism behind enhanced charge transfer at the heterojunction between two-dimensional carbon nitride catalysts (poly-heptazine imide (PHI) and poly-triazine imide (PTI)) during the oxygen reduction reaction. We found that PHI crystallites grow on PTI layers formed at the gas–liquid interface in the salt melt, following the [001]_PTI_/[001]_K‑PHI_ orientation. This crystallographic alignment promotes the charge transfer from PTI to PHI and creates an electron-rich interface. Electron energy loss spectroscopy showed quaternary N atoms in the heterojunction, which aid O_2_ adsorption and 2e^–^ reduction to H_2_O_2_, as well as a higher proportion of terminal and bridging N atoms, promoting charge separation during the reaction.

## Graphical abstract

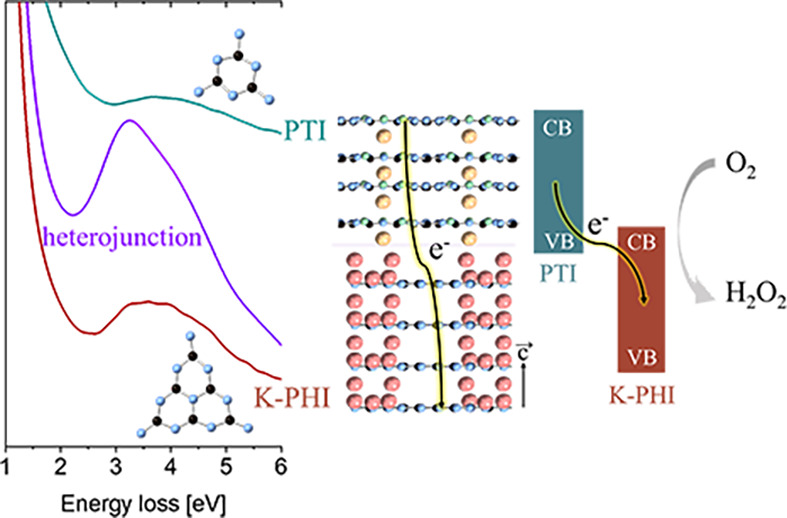

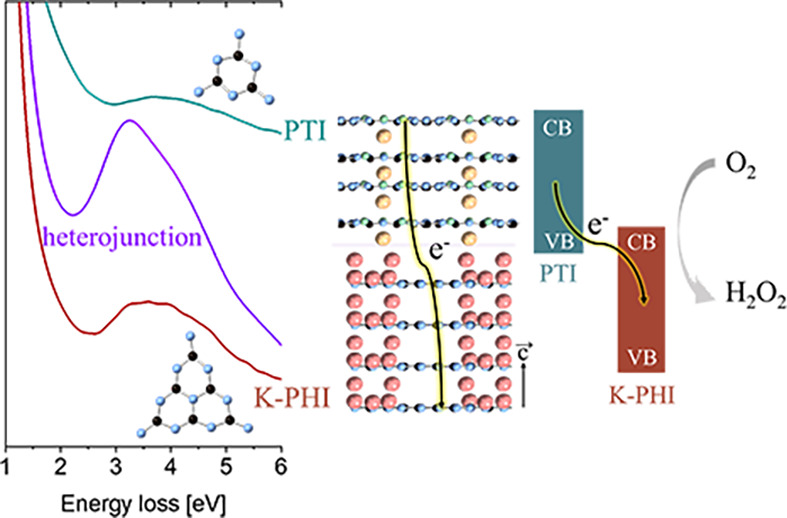

## Introduction

Organic semiconductors are emerging as a new class of compounds to be broadly used in a variety of electronic devices (solar transducers, field-effect transistors, photodetectors, etc.)^1^ owing to their unique combination of desirable qualities such as mechanical flexibility, mixed ionic and electronic conduction, stability of their surface in air, and solution processability.^2^ Many notable organic semiconductors of commercial interest are π-conjugated molecules or polymers, such as conjugated polyphenylenes, polyfluorenes, phthalocyanines, polycyclic aromatic hydrocarbons, and thiophene-containing conjugated polymers, to name a few. The π–π interactions formed between two adjacent organic molecules in such compounds strongly influence functional properties such as the mobility of charge carriers, optical and transport gaps, exciton size, etc. Various materials can be easily assembled together through π–π interactions, forming organic–organic interfaces and becoming an ideal playground to tailor both optical and transport-related properties of organic electronic devices.^3,4^

To be able to precisely control complex structure–property relationships in these compounds and specifically at the organic interfaces, a technique that enables acquiring information about atomic structure, chemical composition, and electronic structure up to a very high spatial resolution is required. Analytical scanning transmission electron microscopy ((S)­TEM) was found to be one of the few methods that perfectly fulfills these criteria, enabling not only imaging of organic compounds/interfaces with nanometer and atomic resolutions^5^ but also combining it with an analysis of chemical compositions, chemical bonding, electronic structure, and optical properties of interfaces using electron energy loss spectroscopy (EELS) and X-ray energy dispersive spectroscopy (EDX).^6,7^ Most publications related to the characterization of organic interfaces are dedicated to photovoltaic structures, where detailed information about phase separation, crystal structure of organic films, and electronic structure of a donor–acceptor interface is critical for understanding the mechanism of exciton dissociation and for improving device efficiency. Several reviews dedicated to this topic are available.^7,8^ One has to mention, however, that scanning transmission electron microscopy for the characterization of technologically relevant organic interfaces is still far from routine due to several challenges, such as (1) susceptibility of the organic materials to electron beam damage, (2) the buried nature of organic interfaces, and (3) the semicrystalline nature of many organic compounds coupled to the low scattering power of light elements. To overcome these issues, the development of specific methodologies and workflows tested on different kinds of organic–organic interfaces is urgently required.

In this work, we explore the application of transmission and scanning transmission electron microscopy to study heterojunctions between two-dimensional carbon nitride catalysts: potassium poly-heptazine imide (K-PHI), C_12_N_17_(H_*x*_K_1–*x*_)_3_[H_2_O]_7_, and poly-triazine imide (PTI), C_12_N_18_(H_*x*_Li_1–*x*_)_3_LiCl. This type of heterojunction outperformed previously reported carbon nitride standards in the oxygen reduction reaction with the production of H_2_O_2_.^9^ We are specifically interested in understanding the nanoscale mechanism behind the enhancement of photocatalytic activity by several orders of magnitude compared to those of the separate constituent phases (K-PHI and PTI). However, using electron microscopy for the characterization of this interface poses several specific challenges. The material is a powder in which both phases consist of strongly connected and intermixed nanocrystals.^9^ In addition, K-PHI and PTI have similar C/N ratios and molecular and crystal structures (Figure 1), which complicates distinguishing them in the mixture. K-PHI consists of heptazine (tri-s-triazine) units connected via negatively charged nitrogen atoms (imide bridges), forming 2D layers. These layers are stacked in an AAA sequence and are bound by π–π interactions. Potassium atoms are located between layers and within channels in the structure, counterbalancing the charge on imide bridges^10,11^ Similarly, PTI also forms a layered structure. In this case, the layers consist of triazine units linked through imide bridges, following an AAA stacking sequence as well. Two adjacent layers are rotated 180° with respect to each other, keeping triazine units aligned, while imide bridges alternate sides.^12,13^ K-PHI and PTI also have similar calculated bandgap values, which are 2.74 and 2.94 eV, respectively.^9^

**Figure 1:**
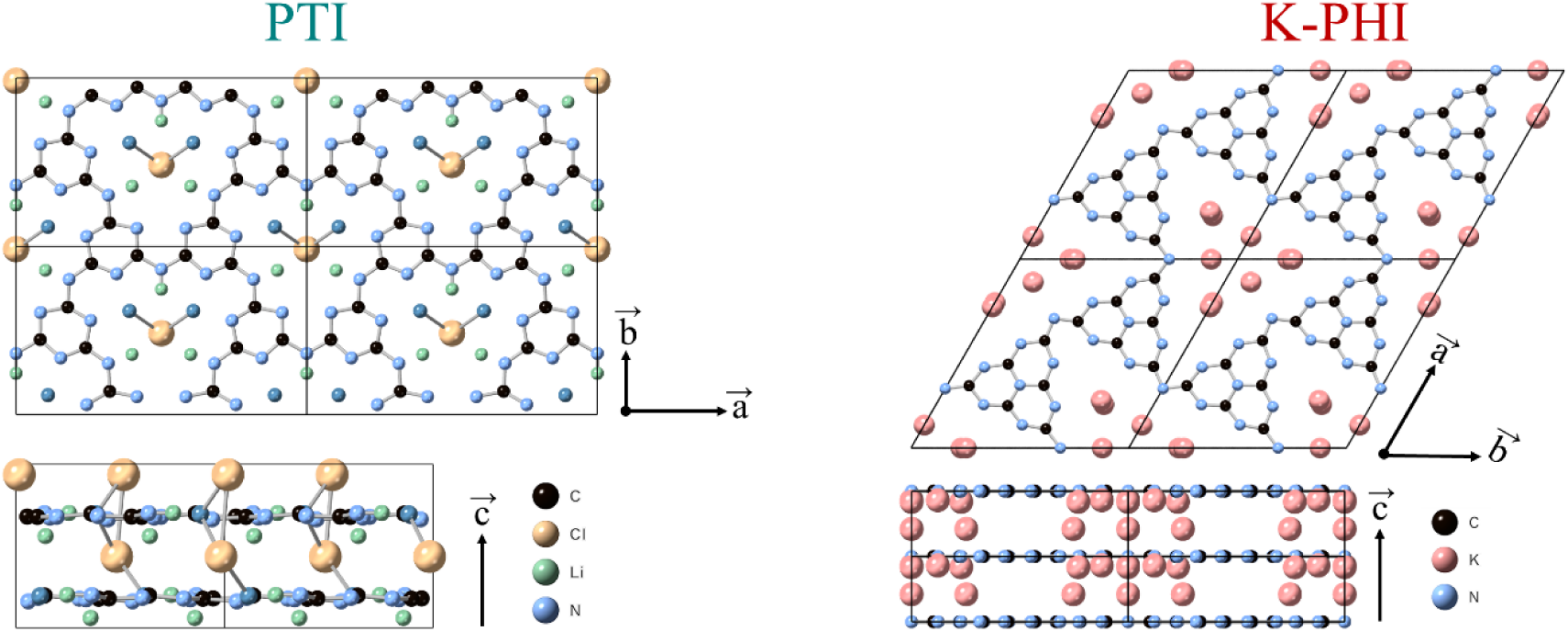
Atomic structures of potassium poly-heptazine imide, K-PHI (right), and of poly-triazine imide, PTI:LiCl (left). Black, blue, yellow, green, and pink spheres indicate carbon, nitrogen, chlorine, lithium, and potassium atoms, respectively.

To distinguish these slight structural and electronic variations and to relate them to catalytic properties, we performed a comparative study of the atomic local environment, bonding, and electronic structure of K-PHI, PTI, and K-PHI/PTI heterojunction samples, combining pair distribution functions (PDFs) analysis,^11,14^ radial distribution functions (RDFs) derived from the extended energy loss fine structure (EXELFS),^15^ and analysis of near-edge fine structure (ELNES) and the valence region of electron energy loss spectra (EELS). While ELNES is commonly used to characterize organic compounds, the analysis of valence EELS and PDF^16^ in organic compounds is still relatively rare, and just a few papers refer to EXELFS-derived RDF studies involving light elements,^9,17,18^ making our approach unique. Figure 2 summarizes the workflow and the type of information that we obtained from each of the employed methods.

**Figure 2:**
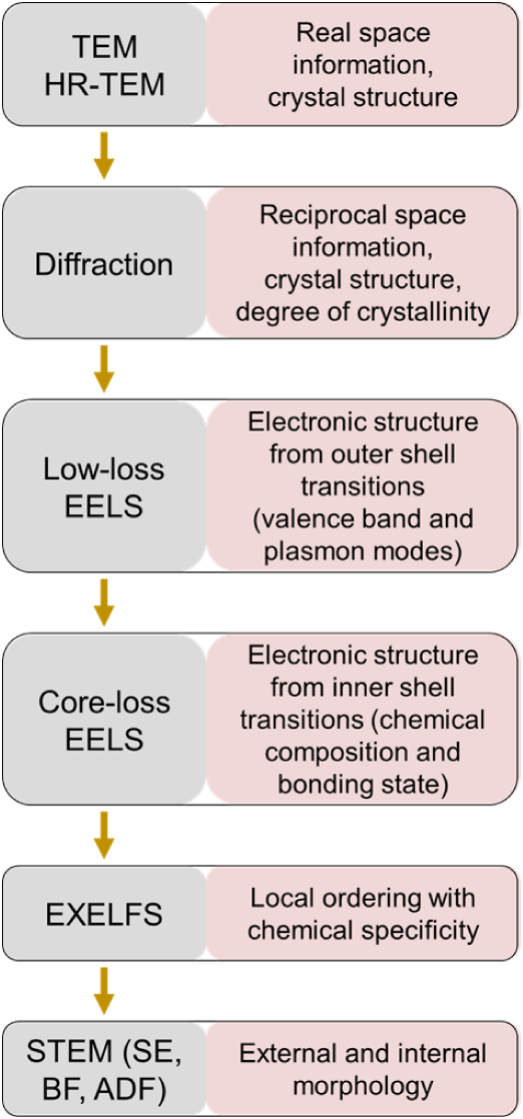
Schematic representation of the workflow used in this study and a summary of the information provided by each technique.

## Results and Discussion

### Morphology, Crystallinity, and Structural Alignment of Heterojunction’s Constituent Phases

Overview TEM and HR-TEM images of PTI, K-PHI, and heterojunction samples are shown in Figure 3. Each of the obtained compounds exhibits a clear and distinct morphology. PTI forms micrometer-sized, curved (sometimes tubular) flakes, which consist of crystalline grains 9.7 ± 1.6 nm in size (Figure 3a). Such morphology has been observed frequently in PTI samples before^19^ and has been attributed to the directional growth of crystalline domains on the salt as the substrate. The analysis of Fast Fourier Transforms (FFTs) obtained from different grains in HR-TEM images shows that all of the FFTs can be indexed in an orthorhombic unit cell with lattice parameters *a* = 14.71(10) Å, *b* = 8.50(5) Å, *c* = 6.66(5) Å, confirming the formation of PTI^13^ (Table 1). This result corroborates well with the analysis of the SAED patterns obtained from micrometer-sized flakes and with the analysis of synchrotron-based X-ray diffraction profiles (Figures 2SI and 3SI). The K-PHI sample consists of rod-shaped crystallites with a width of 28.2 ± 9.5 nm and a length of more than 100 nm, agglomerated into larger flakes. An accurate measurement of the crystallites length is not possible due to strong agglomeration that does not allow for differentiating where the edges of each crystallite are located (Figure 8SIa). The observed morphology differs from the typical appearance of K-PHI agglomerates. The analysis of FFTs from HR-TEM images and SAED patterns confirmed that K-PHI crystallizes in a hexagonal unit cell with lattice parameters *a* = 12.66(5) Å, *c* = 3.30(5) Å as described by Savateev et al.^10^ Unit cell parameters are given in Table 1. The heterojunction (HEJ) sample consists of micrometer-sized flakes decorated with rod-like crystallites 11.8 ± 1.8 nm wide (Figure 3c). The FFTs of these crystallites were indexed by using the K-PHI unit cell (Table 1). Figure 3d shows the flat HEJ sample with K-PHI crystals grown on the PTI layer along the viewing direction. FFTs of PTI and PHI crystallites, as well as from a bigger area, show reflections of {100} K-PHI planes and {200} PTI planes, indicating a structural orientation relation close to that of [001]_PTI_/[001]_K‑PHI_.

**Figure 3:**
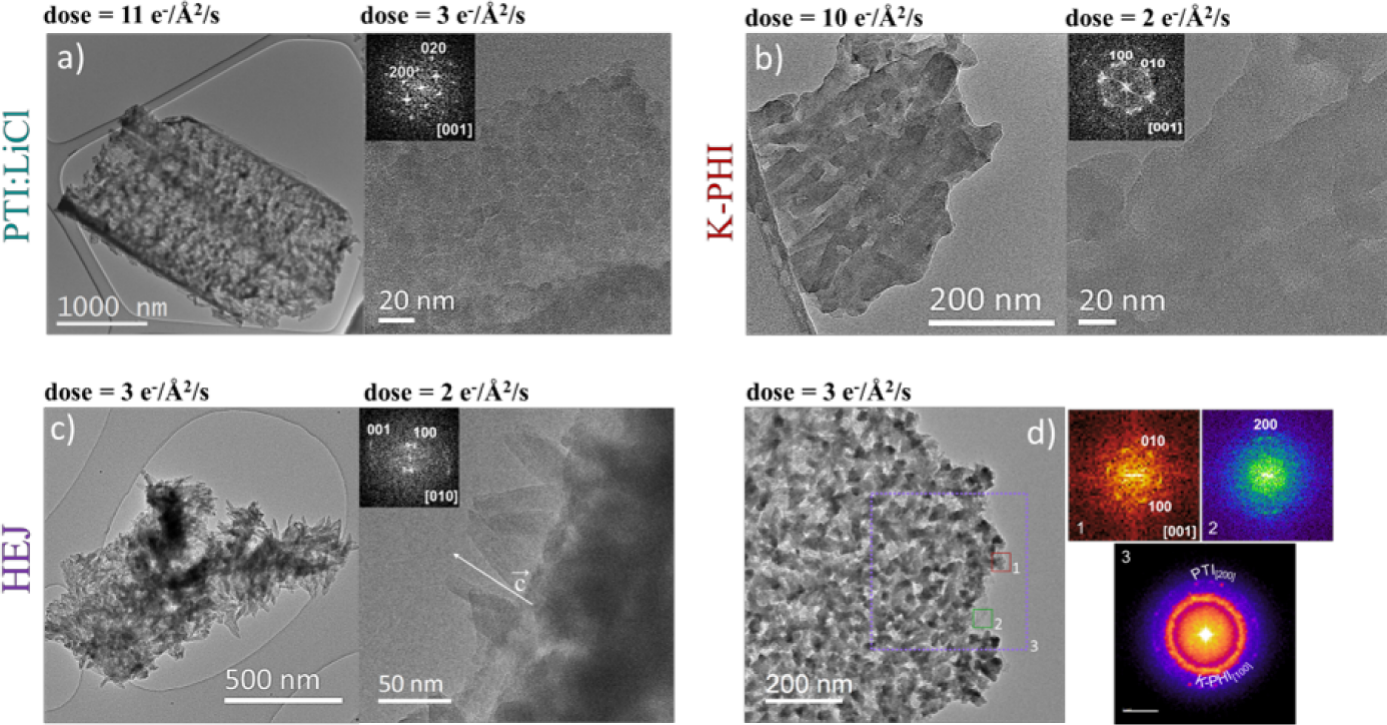
(a–c) Overview (left) and HR-TEM (right) images of PTI, K-PHI, and HEJ with dose rates given above each image. FFTs obtained from the corresponding HR-TEM images are shown in insets. (d) HEJ sample showing the flat flakes decorated with K-PHI growing on PTI with an orientation relation close to [001]_PTI_/[001]_K‑PHI_ inferred from FFTs analysis (1—K-PHI crystallite, 2—PTI crystallite, and 3—bigger area of the flake).

**Table 1:** Comparison of the Main Crystallographic Features of the Three Materials

	PTI	K-PHI	HEJ
Morphology	Micrometer-sized tubular shaped flakes	Rod-shaped crystallites agglomerated into micrometer-sized flakes	Micrometer-sized flakes decorated with rod-like K-PHI crystallites
Grain size^a^	9.7 ± 1.6 nm	W: 28.2 ± 9.5 nm	W: 11.8 ± 1.8 nm (for K-PHI)
		L: >100 nm	
Crystal system	Orthorhombic	Rhombohedral	
	*Cmc*2_1_	*P*31*m*	
Unit cell parameters [Å] obtained from SAED data	*a* = 14.71(10)	*a* = 12.66(5)	K-PHI: *a* = 12.64, *c* = 3.30
	*b* = 8.50(10)	*c* = 3.31(5)	PTI: *a* = 14.68, *b* = 8.48, *c* = 6.65
	*c* = 6.66(10)		
Unit cell parameters [Å] obtained from sPDF data	*a* = 14.62(10)	*a* = 12.66(5)	K-PHI: *a* = 12.63, *c* = 3.30
	*b* = 8.44(10)	*c* = 3.30(5)	PTI: *a* = 14.67, *b* = 8.48, *c* = 6.65
	*c* = 6.62(10)		

a Measured from HR-TEM images.

The azimuthally averaged scattering profiles obtained from SAED patterns for all three samples are shown in Figure 2SI. The scattering profile obtained from the heterojunction displays peaks corresponding to both K-PHI and PTI. Unfortunately, due to strong variations in the thickness of the flakes and a limited *Q*-range (up to 7.5 Å^–1^) of the recorded SAED patterns (a hardware limitation of our system), we could not obtain reliable electron pair distribution functions (ePDFs). This is why, to gain a deeper understanding of the structure at the interface, we collected synchrotron pair distribution functions­(sPDFs) of all three samples. Figure 4 shows the sPDF results and structural models, with the main atomic pairs marked. For simplicity, we show pairs with N atoms taken as central scattering atoms, allowing for a direct comparison of these data with the N-edge EXELFS-derived RDF, which are discussed in detail later in the text. The sPDF obtained from HEJ is mainly dominated by the K-PHI signal, especially the distances above the 4 Å range, corresponding to atomic pairs between atoms from adjacent layers. The small peak at 1.85 Å is assigned to the N–Me distance.^11,20^ Analysis of the scattering profiles in reciprocal space (Figure 3SI) confirms the presence of both phases in the heterojunction material. The peaks appear broader compared to pristine K-PHI and PTI, indicating a slightly decreased crystallinity of the HEJ sample. The most intense scattering signals come from the {001}_K‑PHI_ and {002}_PTI_ sets of planes. We observed several sharp reflections in the scattering profiles of pure PTI that can be indexed using the face-centered cubic unit cell of LiCl. Most probably, the alkali metal salt could not be fully removed during the washing step of the synthesis procedure. We did not observe any reflections from additional phases in the scattering profiles from the heterojunction and K-PHI.

**Figure 4:**
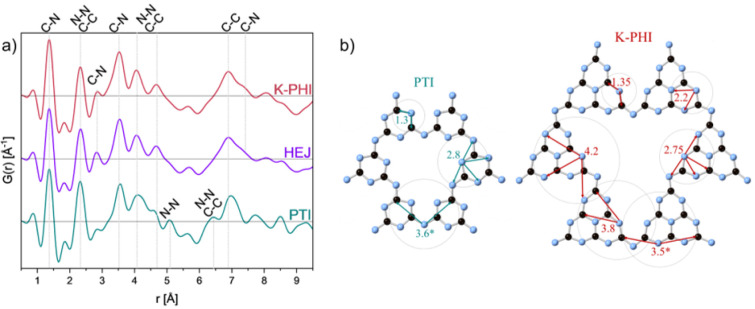
(a) sPDFs of K-PHI (red), PTI (green), and heterojunction (purple) with corresponding atomic pairs marked. (b) Projections of the crystal structures of PTI and K-PHI along the c-direction with atomic distances marked in green and red. *Corresponds to distances between atoms of adjacent layers as well.

Thus, from the analysis of SAED, sPDFs, and crystallographic directions and morphology of the crystals on HR-TEM images, we conclude that both constituents of the HEJ material are crystalline, and their crystallographic *c*-directions are aligned. The observed match between the width of K-PHI crystallites in the heterojunction and PTI crystallites (calculated from the pure phase) is a further indication for this crystallization preference. It is worth mentioning that previous reports on carbon nitrides suggested that crystallographic alignments of different phases should promote photogenerated charge mobility.^9^ In particular, upon dissociation of excitons, charge migration between neighboring heptazine units is sluggish, resulting in a higher probability of recombination events to occur. We suggest that the alignment of both semiconductors along the *c*-direction^21,22^ is probably one of the factors responsible for improved mobility of photogenerated e^–^ and h^+^ through both layers.^9^

To understand the morphology of the heterojunction sample, we compared STEM images recorded using bright-field (BF) and annular dark-field (ADF) detectors with the corresponding secondary electron (SE) images. SE-STEM images of the heterojunction material show flakes with flat surfaces forming pocket-like structures with K-PHI crystals growing inside. The latter are clearly visible in ADF-STEM and BF-STEM images (Figure 5). The observed morphology is crucial for understanding the formation mechanism of the heterojunction, discussed in detail below.

**Figure 5:**
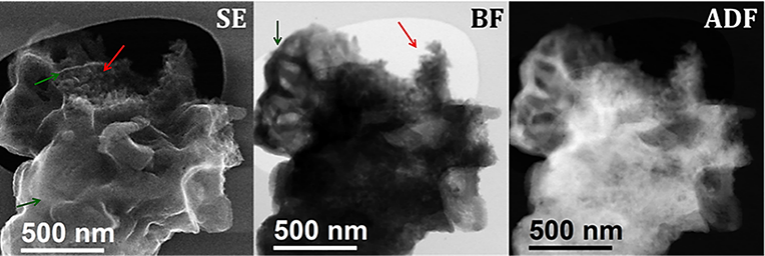
Secondary electrons (SE), bright field (BF), and annular dark field (ADF) STEM images of the HEJ material showing the smooth pocket-like morphology (green arrows) with K-PHI crystallites growing inside these pockets (red arrows).

### Spatially Resolved Electronic Structure and Chemical Composition

#### Low-Loss EELS to Probe Valence Electron Transitions at Heterojunction

The low-loss region of an EELS spectrum (often referred to as valence EELS (VEELS)) contains features associated with the excitation of the valence electrons of a solid, in particular interband transitions and plasmons.^23–25^ Figure 6a presents the VEELS spectra obtained from the three materials with two distinct spectral features, the π-plasmon and bulk plasmon contributions. In the case of the K-PHI and the PTI samples, π-plasmons appear as broad peaks with long tails. K-PHI has an onset at 2.5 eV and two maxima at about 3.25 and 4.3 eV. The PTI signal has an onset at 2.9 eV with a maximum intensity peak at 4.1 eV and a more complex structure of the bulk plasmon. In the heterojunction sample, the π-plasmon has an onset at 2.3 eV and consists of two peaks with maxima at 3.25 and 4.2 eV, while the bulk plasmon reflects the presence of several excitations. From this qualitative VEELS analysis, it is inferred that the surface electronic structure of the heterojunction is mainly influenced by the K-PHI phase, while the bulk excitations are largely affected by the PTI phase. To gain more accurate insight into the surface electronic structure, aloof VEELS spectra were collected (Figure 6b). Bandgap energies, calculated using the intersecting lines method^15,25^ on the π-plasmon peaks, were found to be 2.7 eV for K-PHI, 2.5 eV for the heterojunction, and 3.0 eV for PTI, which are in good agreement with earlier reported values for optical band gaps in these materials.^9,26^

**Figure 6:**
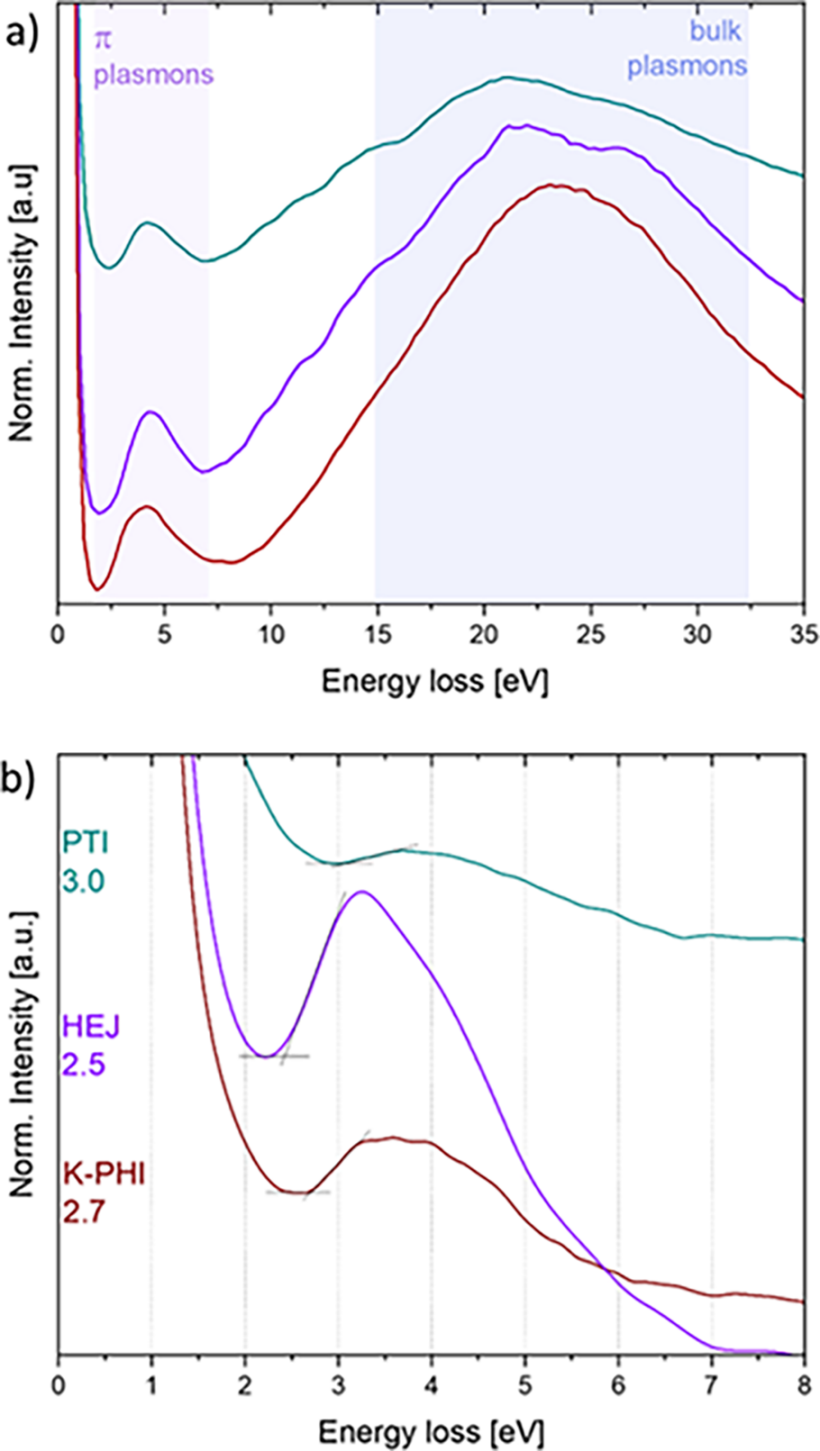
VEELS spectra of K-PHI (red), HEJ (purple), and PTI (cyan) collected (a) with the electron beam positioned on particles and (b) with an aloof beam; the band gap values are indicated on the left.

Under nearly identical collection conditions and at very similar sample thicknesses (Tables 2SI and 3SI), we observed very different overall signal intensities in the aloof EELS spectra. The PTI shows a significantly lower response compared to the other samples, which can be partially attributed to the structure of this material, particularly the presence of grain boundaries or more electronegative atoms on the surface. The HEJ sample exhibits the most intense response, with a definite maximum at 3.25 eV, as well as a slightly narrower band gap value. Such a π-plasmon response might be pointing toward the creation of a higher population of charges upon semiconductor excitation compared to pure phases, which is a consequence of a higher degree of charge separation between the two components of the investigated heterojunction.

To map the π-plasmon contributions of each component in the heterojunction, we recorded the VEELS signal from the whole particle. One can see a change in the intensity ratio between the two contributions to the π-plasmon when we record in aloof mode and when we place the beam on the particle. By fitting the π-plasmon peak with two pseudo-Voigt functions, we were able to separate the contributions of the two components and precisely locate them in the VEELS map of the heterojunction (Figure 7). A peak at about 3.4 eV corresponds to the signal coming from K-PHI crystals, while the main intensity of the 4.3 eV peak is localized at the interface between K-PHI and PTI. Figure 7e shows the mixed signals. We observe a white rim exactly at the interfaces where K-PHI and PTI meet, pointing toward an enhanced signal compared to the rest of the spectroscopic map. EELS spectra extracted from different regions of the particle were compared to check the π-plasmon response, and an increased signal intensity was observed compared to the other analyzed regions (Figure 4SI). Using this approach, we can obtain a better view of the electronic structure at the interface between K-PHI and PTI, which is particularly useful when a clear cross-sectional view of the interface is not possible to obtain in the TEM images.

**Figure 7:**
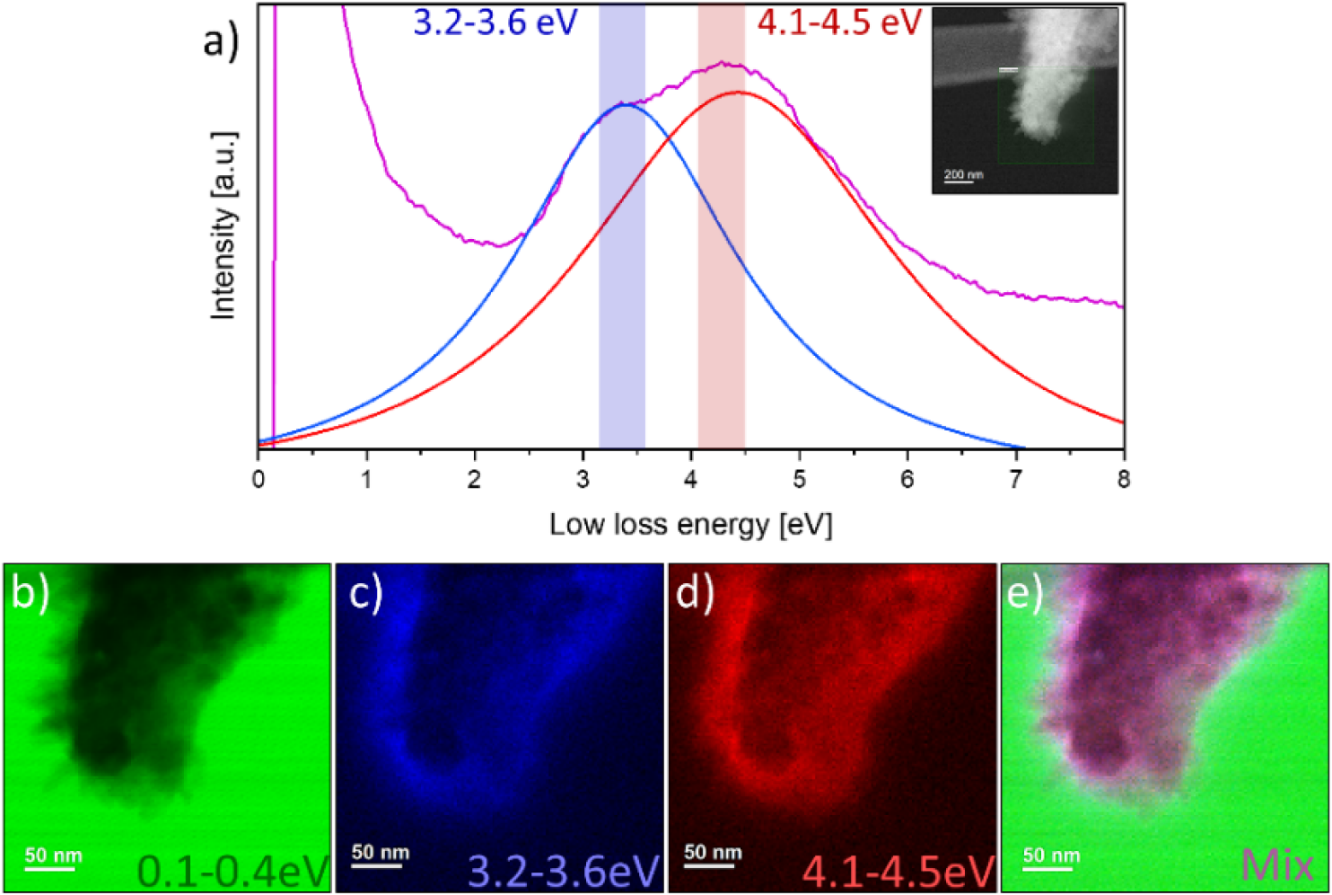
(a) π-plasmon peak fitted with two pseudo-Voigt functions and spectral windows used for mapping: 3.2–3.6 eV (blue) and 4.1 −4.5 eV (red), (b–d) corresponding plasmon maps, showing the formation of (e) an electronic interphase seen as a white thin region at the edge of the particle.

### ELNES Analysis to Probe Defects and Adsorption Sites at Heterojunction

Chemical and bonding environments were investigated at the nanoscale using ELNES analysis. Figure 8 shows EELS spectra, with the carbon and nitrogen K-edges recorded from all three compounds. By calculating the second derivative,^27^ we can precisely identify the ionization energy (*E*_0_) of C and N to be at 285 eV and 399.3 eV, respectively, in all studied compounds. The C 1s-π* transitions appear as several peaks in the spectra. The signal at 288.1 eV corresponds to carbon atoms bound to bridging or terminal nitrogen in all three samples (denoted as C_2_). In the spectra from K-PHI and the heterojunction, the 287.0 eV signal is attributed to sp^2^—hybridized carbon in imide rings (C1), while the shoulder at 286.2 eV is linked to CH_*x*_ groups. In the PTI phase, a unique signal at 286.6 eV is assigned to surface-bound C–O or C=O functional groups. The C 1s-σ* transitions appear at approximately 297.7 and 300.1 eV. Additionally, we observed the L_2,3_ edges of potassium at 294 and 296 eV, respectively. For all three materials, N 1s-π* signals are detected at 400.4 and 401.8 eV, representing nitrogen atoms within imide rings (N_1_) and bridging or terminal groups (N_2_). Quaternary nitrogen (N_3_), bound to carbon in heptazine units, appears as a shoulder at 402.8 eV in the spectra from K-PHI and the heterojunction. All spectral values and assigned chemical bonding are summarized in Table 4SI. The presence of these atomic species is crucial for photocatalytic O_2_ reduction and the possible mechanistic pathway such a process can take. N_3_ centers allow an end-on adsorption mode of O_2_, thus favoring a 2e^–^ process and yielding H_2_O_2_ as the final product. On the other hand, adsorption on PTI would occur between two neighboring triazine units in a side-on fashion, thus leading to a dissociative mechanism which yields H_2_O.^28^

**Figure 8:**
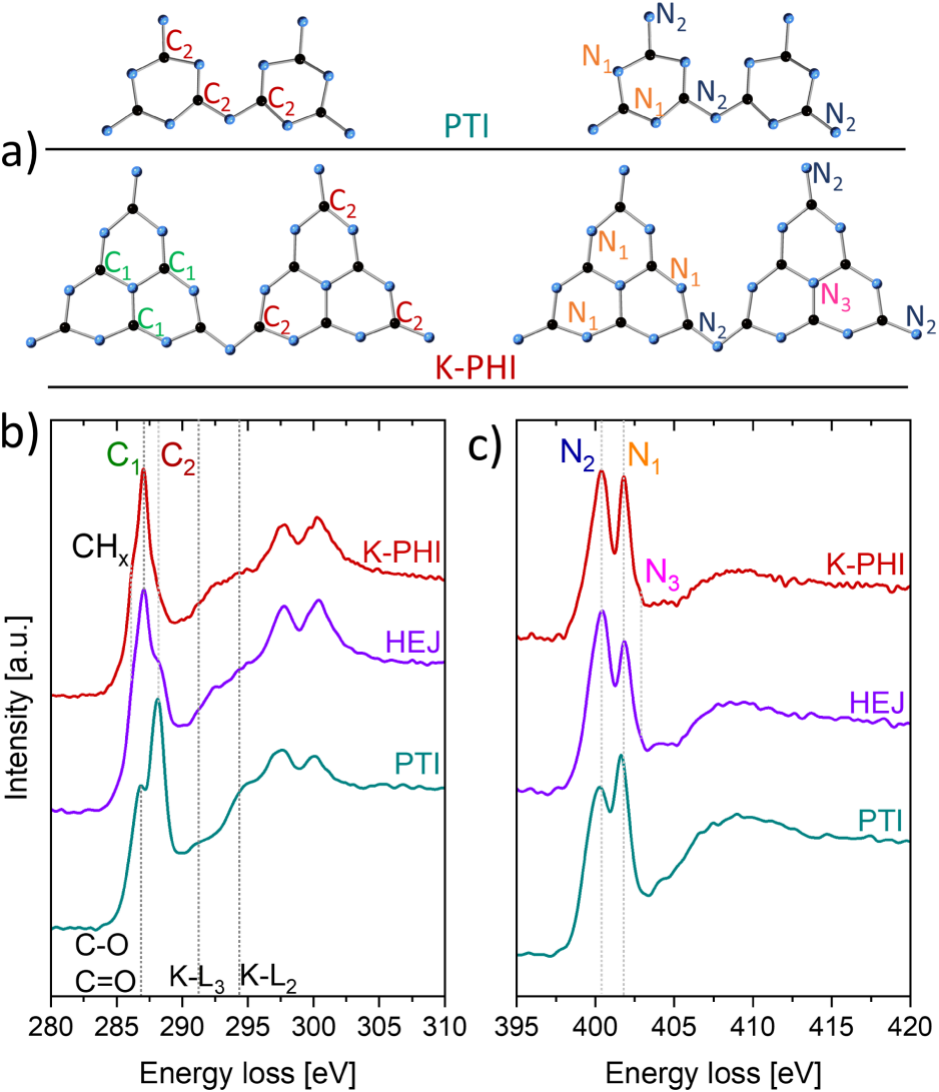
(a) Structure of triazine or heptazine 2D layers in PTI and K-PHI with different C and N atoms marked as C1—green, carbon atoms bonded to 3 N atoms in the imide ring, C2—red, carbon atoms bonded to 3 N atoms, one being bridging or terminal, N1—orange, imide ring atoms, N2—blue, terminal and bridging atoms, and N3—quaternary atoms. (b, c) ELNES of the C and N K-edges of K-PHI (red), heterojunction (purple), and PTI (cyan) with assigned chemical bonding.

Therefore, in both K-PHI and HEJ, the presence of these quaternary nitrogen atoms is a key factor that allows photocatalytic H_2_O_2_ evolution to occur. The N 1s-σ* features, however, are less well-defined compared with those of the C K-edge. Based on these data, we calculated intensity ratios between N_1_/N_2_ signals, N/C signals, and the sp^2^/sp^3^ ratio for carbon and nitrogen. We point out that the intensity value of the ELNES signal indicates a probable transition between the 1s occupied core level and the unoccupied levels in the antibonding orbitals (π* or σ*), which can be used to estimate the proportion of specific atomic moieties present in the samples. From these calculations, we conclude that in PTI, the N_1_ moieties (atoms within imide rings) and sp^2^-hybridized carbon contributes more to the spectra, reflecting the higher degree of crystallinity within the PTI layers, in agreement with the HR-TEM and diffraction results. In contrast, in K-PHI, carbon sp^3^- and sp^2^-contributions are nearly equal, pointing toward the presence of defects in the K-PHI crystals. The C/N ratio is the same in both K-PHI and heterojunction samples, with the content of C being slightly lower than that of N. The heterojunction’s chemical structure contains the highest proportion of N_2_ moieties (bridging and terminal nitrogens) and the highest proportion of sp^3^-hybridized carbon. The observed increase in bridging and terminal N atoms (N_2_ moieties) compared to the other samples suggests that the heterojunction contains a higher concentration of defect sites with unpaired electrons. This explains the enhancement of charge transfer to the O_2_ atoms adsorbed at the N_3_ atoms during the oxygen evolution reaction.^28^ We would like to mention that the presented calculations help to estimate the trend in the samples’ bond structure; to obtain a quantitative description of the C- and N-hybridizations, the integral intensity ratios of 1s-π* and 1s-σ* peaks from the spectra measured at the so-called magic angle in TEM (at which the measured spectrum becomes independent of the sample’s orientation relative to the electron beam) have to be obtained and compared with the corresponding spectra of the standards (e.g., the standard with 100% C in sp^2^ hybridized states or with 100% of N in sp^2^-hybridization).^29–31^

### EXELFS to Derive Local Ordering Around N Atoms

Radial distribution functions (RDFs) were derived from the EXELFS region of the N K-edge for both the K-PHI and heterojunction samples (Figure 9b). The bond lengths of approximately 1.3 Å, 1.9 Å, 2.2 Å, 2.75 Å, 3.35 Å, and 3.5 Å are resolved on the experimental RDFs of both compounds. They are in good agreement with sPDF data (see the corresponding section above) and interatomic distances typically found in carbon nitrides (Table 2). However, the intensities of the peaks on RDFs differ considerably between the K-PHI and HEJ samples. The O K-edge signal at 532 eV in the PTI spectrum prevented us from obtaining the N K-edge experimental RDF of PTI (Figure 9a). The spectral images of the PTI sample (Figure 7SI) show that the signal from the O K-edge is mostly localized at the edges of the flakes, suggesting that oxygen has either been absorbed on the surface or formed C–O and C=O functional groups. The latter observation corresponds well with the ELNES structure of the C K-edge of PTI. Theoretical calculations of the nitrogen RDF of K-PHI were obtained using the *ab initio* FEEF10 code and the structural model proposed by Savateev et al.^10^ (sp. gr. *P*31*m*) as input. Peak positions and peak intensities of the experimental and theoretical RDFs of K-PHI (Figure 9b,c) are in good agreement. Two PTI structural models proposed by Liao et al.^13^ (sp. *Cmc*2_1_) and Wirnhier et al.^12^ (sp. gr. *P*63*cm*) were used for calculations of theoretical RDFs.

**Table 2:** Atomic Distances Obtained from RDFs (N K-Edge EXELFS Spectra) and Corresponding Atomic Coordination

Atomic distance [Å]				
PTI Li:Cl^a^	K-PHI^b^	HEJ^b^	Coordination	
1.3	1.35	1.3	N–C	In plane
1.85	1.9		N–Li/N-K	
2.5		2.2	N–N	
2.8	2.75	2.75	N–C	
3.2	3.35	3.35	N–N	Adjacent layers
3.6	3.5	3.5	N–C	
	4.2	4.15	N–N	

a Calculated from theoretical RDF.

b Calculated from experimental RDF.

**Figure 9:**
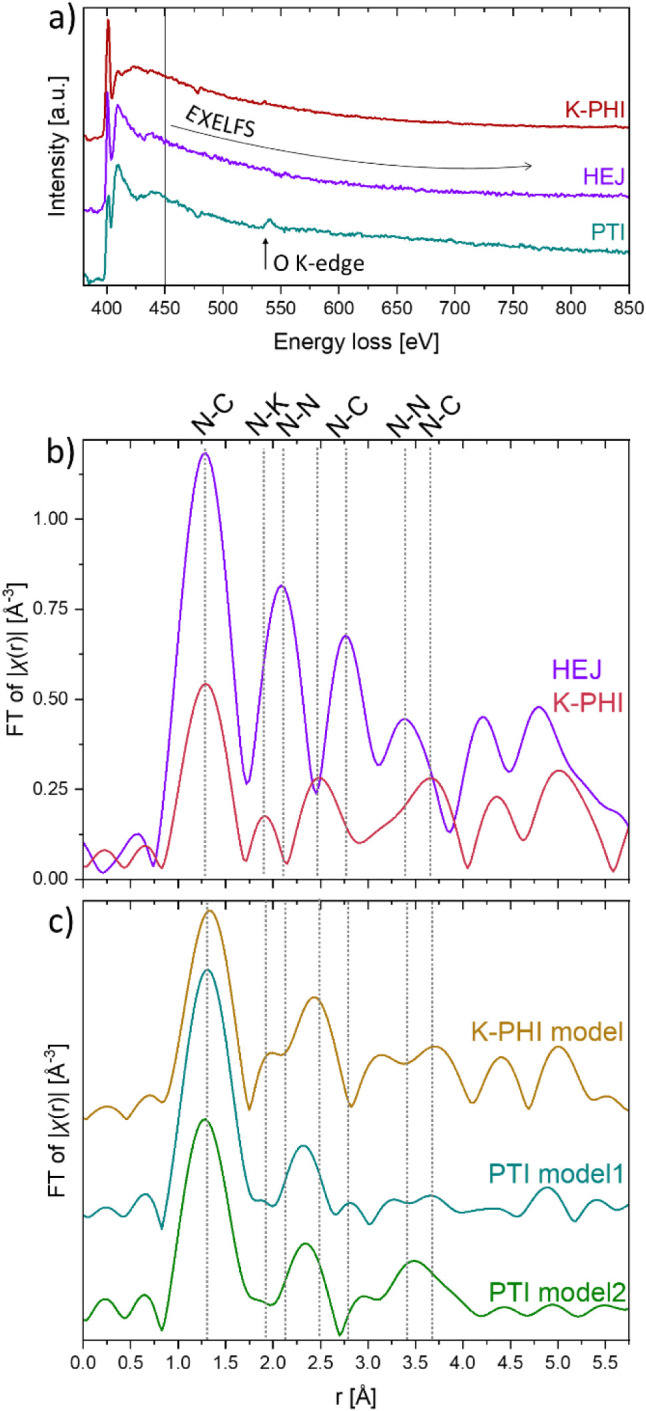
(a) Nitrogen K-edge EXELFS of all three samples with the O K-edge on the PTI spectrum. (b) Corresponding RDFs (red—K-PHI and purple—HEJ). (c) Theoretical RDFs obtained from two PTI models with different crystal symmetries: PTI model 1 −2-fold rotation axis (dark cyan) and PTI model 2–6-fold rotation axis (green).

The intensities of the peaks are strongly influenced by the model’s symmetry. The RDF obtained using Model 2, which has high crystallographic symmetry, influences the intensities of the distances between atoms of adjacent layers, showing an increased intensity compared with Model 1 (Figure 9c). The latter is in good agreement with electron diffraction data and, when combined with the K-PHI model, also provides a better match to the RDF of the heterojunction. It is observed that in the RDF from HEJ, the second peak, which corresponds to N-Me and N–N distances, displays an increased intensity compared to the N-Me/N–N distances from the individual phases. This corresponds well with the higher proportion of terminal and bridging N atoms observed in the ELNES spectra from the heterojunction; however, we cannot define the exact origin of this increase.

### Mechanism of Heterojunction Formation

A detailed analysis of the crystal and electronic structures of the heterojunction, along with its morphology observed from STEM images, is combined with extensive analysis of the thermal behavior of poly-heptazine and poly-triazine imide precursors^32,33^ in salt melts. This enables us to propose a structure of the interface between the K-PHI and PTI Li:Cl phases and to suggest a possible mechanism for the formation of this heterojunction.

A review of the thermochemistry of the precursors^34,35^ and the thermal behavior of poly-heptazine and poly-triazine imides^32,33^ in salt melts suggests that the annealing process involves the following steps: (1) the KCl:LiCl eutectic salt melts at 352 °C; (2) ring-opening reactions of 5-aminotetrazole (5-AmT) occur between 290 and 520 °C, releasing gaseous byproducts such as NH_2_CN (cyanamide), HCN (hydrogen cyanide), NH_3_ (ammonia), N_2_ (molecular nitrogen), and HN_3_ (hydrazoic acid). So, one can notice that gas phases are formed almost throughout the entire annealing process; (3) at around 300 °C melamine begins to condense, forming heptazine units at 390 °C and releasing NH_3_ gas, with oligomer formation occurring above 400 °C. The next three stages happen at temperatures between 520 and 550 °C: (4) at 520 °C, heptazine units polymerize into melon; (5) at 530 °C, the melon polymeric structure breaks down into poly-triazine imide units (PTI); (6) while at 550 °C, the K-PHI structure is formed from the gaseous product of 5-AmT decomposition. Note that PTI forms from melon units present in the salt melt, while K-PHI forms from the gaseous precursors that are not solubilized in the KCl salt melt. This difference in solubility plays a critical role in the formation of the heterojunction. The presence of undissolved gaseous precursors leads to the formation of gas bubbles. As the temperature rises, melamine condenses into heptazine units, oligomers, and eventually polymeric melon, creating layers less permeable to the gases and thus confining them in pockets (Figure 10). The heptazine units in this layer then dissociate into triazine units, forming a layer composed of nanometer-sized grains of PTI. Grain boundaries formed in the layers of the pure PTI phase are clearly visible in HR-TEM images (Figure 3). These grain boundaries have numerous defect sites where atmospheric oxygen is likely adsorbed when the material is exposed to air. The latter corresponds well to signs of C=O and C–O groups observed in the EELS spectra of PTI (Figure 8). In the case of the HEJ sample, the PTI layer present in the salt melt, covering a pocket of reactive gases, also contains defects. At 550 °C, the gaseous precursors start to polymerize into heptazine units and condense at the defect sites of the PTI surface, forming polycrystalline structures intercalated with K^+^ ions. The latter is supported by the fact that the size of the inward-grown K-PHI crystallites matches the size of the PTI grains in the heterojunction, suggesting that the growth of K-PHI is influenced by the PTI substrate. We also did not observe oxygen adsorption on the surface of HEJ. As mentioned earlier, the analysis of the FFTs obtained from HR-TEM images of K-PHI crystals in the heterojunction reveals that the orientation of the *c*-axis of K-PHI crystals (the direction along which the layers are stacked on top of each other) is close to or parallel to the layer stacking direction of PTI (Figure 3). The proposed growth mechanism explains very well the observed core–shell morphology and the crystallographic relationship of the phases in the heterojunction, where we see that K-PHI crystallites are grown on the PTI substrate with pockets, exhibiting an orientation relation close to that of [001]_PTI_/[001]_K‑PHI_ (Figures 3 and 10). Thus, the presence of defect sites and the alignment of the *c*-axes of both lattices help to create an electron-rich interface and to promote a more efficient charge separation and mobility, corresponding well with the observed decrease in the band gap value and a considerable reduction of the photoluminescence signal from the heterojunction compared to pure PTI and K-PHI.^9^

**Figure 10:**
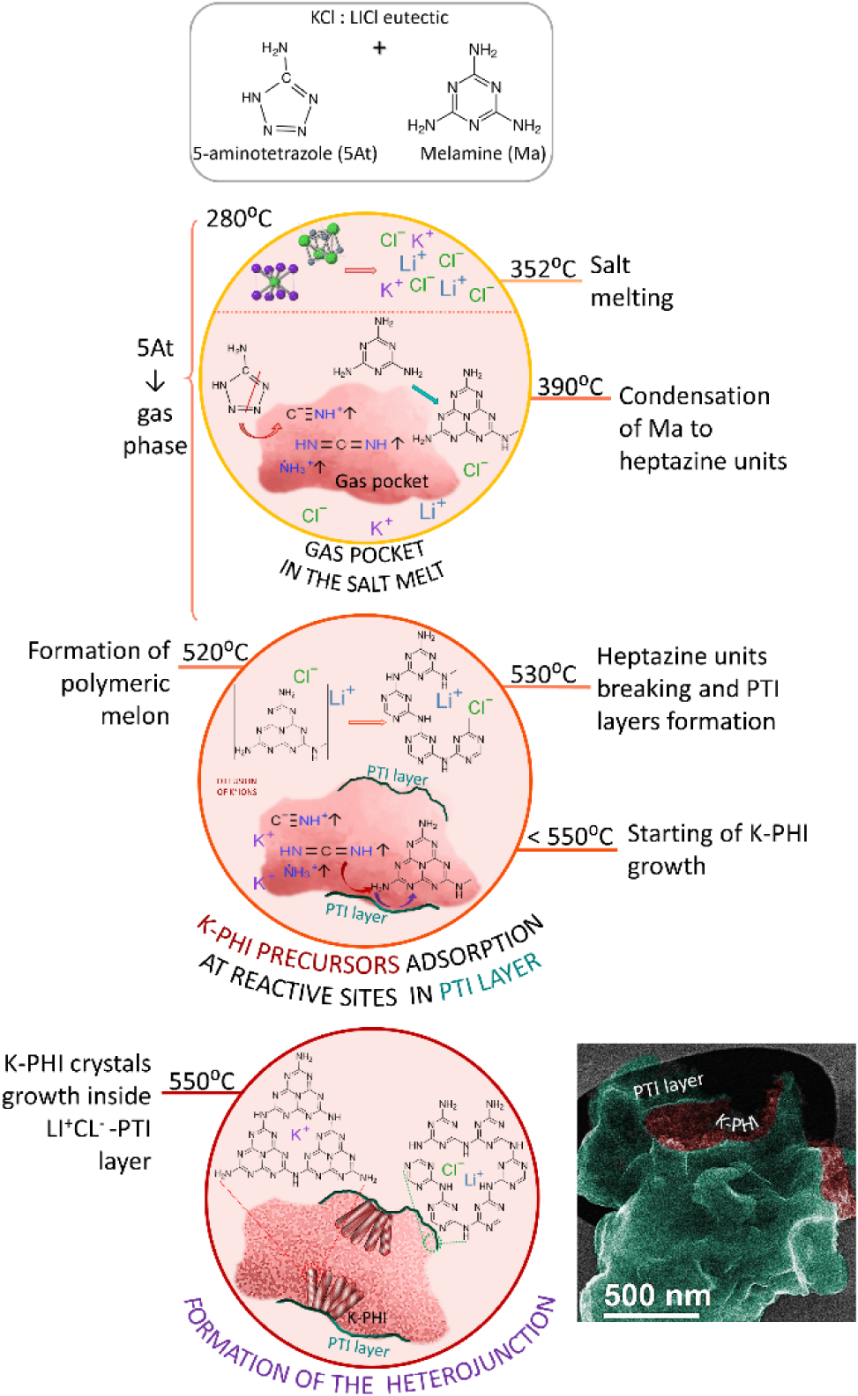
Schematic representation of the mechanism of heterojunction formation, highlighting the main steps of the process. The SE-STEM image with digitally added colors at the bottom of the scheme shows the smooth PTI surface (cyan) of the pocket-like particle and the K-PHI crystallites growing inside (dark red).

## Conclusions

In this work, we performed a detailed nanoscale characterization of the K-PHI/PTI heterojunction with the aim of explaining the enhancement of its photocatalytic activity in the oxygen evolution reaction compared to pure K-PHI and PTI phases. From structural and morphological analysis using HR-TEM, SE- and ADF-STEM, we found that PHI crystallites grow on PTI layers formed at the gas–liquid interface within the salt melt. The orientation relation between the K-PHI and PTI phases is approximately [001]_PTI_/[001]_K‑PHI_. From the analysis of chemical compositions, chemical bonding, and electronic structure using valence and core-loss EELS, we found that the heterojunction contains a significant amount of defect sites and has a narrower band gap (2.5 eV) compared to the pure constituents. The structural alignment of PTI and K-PHI and the presence of defect sites lead to the formation of an electron-rich interface, which promotes an enhanced exciton response. Combining our findings with an extensive analysis of the thermal behavior of poly-heptazine and poly-triazine imide precursors, we were able to suggest the mechanism of heterojunction formation. A key component of our analysis is the combination of different types of imaging modes with detailed spectroscopic analysis inside the scanning transmission microscope, which not only enabled detailed characterization of the buried interface between two materials having similar crystal structure and chemical composition but also helped us to relate their nanoscale structure to their photocatalytic performance. Figure 11 summarizes the main steps in the workflow that we used, highlighting the findings provided for the PTI/K-PHI heterojunction. We believe that the proposed workflow (Figures 2 and 11) can be broadly applied to the analysis of interfaces between organic materials (e.g., interfaces in organic light-emitting diodes, organic solar cell structures, organic sensors, and organic components of wearable electronics) and specifically in cases where the preparation of a TEM sample with a clear cross-sectional view at the interface is not possible. We believe that one of the main challenges with the application of this workflow to different organic interfaces is the development of reliable and artifact-free sample preparation protocols that enable to obtain electron-transparent specimens (e.g., focused ion beam lamellas or ultramicrotomy thin sections). As many organic materials are semicrystalline, we see that another challenge lies in collecting crystallographic data locally, directly from the interface (inside a TEM); therefore, the ability to perform energy-filtered diffraction experiments and to collect SAED patterns up to high Q-ranges (∼20 Å^–1^) is important. Finally, the ability to perform experiments under cryogenic conditions was a very important factor that enabled us to reduce the electron beam damage on the PTI/K-PHI heterojunction during the acquisition of EELS maps, so establishing the measurement conditions that would enable us to avoid damaging the samples by the electron beam is another important experimental challenge.

**Figure 11:**
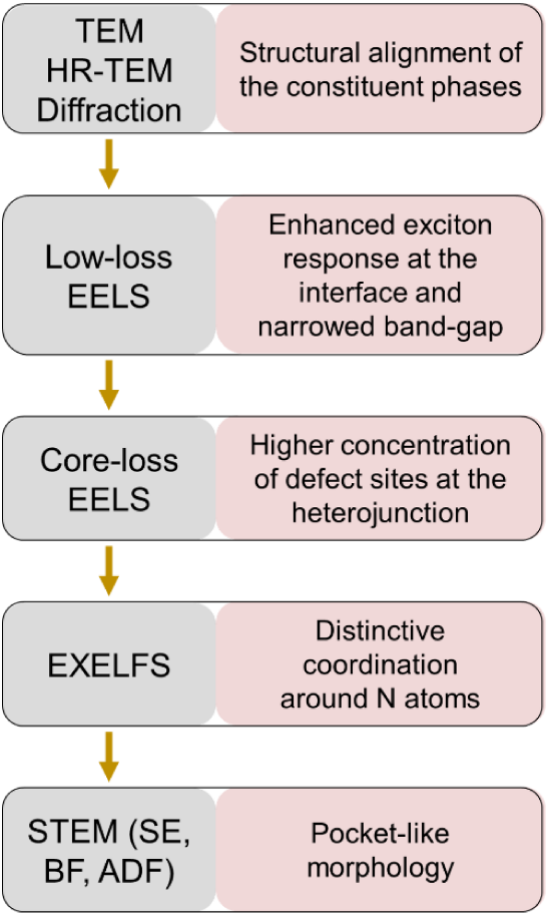
Schematic representation of the workflow used in this study and the main findings obtained from each technique during the characterization of the PTI/K-PHI heterojunction.

## Materials

PTI Li:Cl was obtained from 1 g of melamine (Ma) and 10 g of lithium chloride (LiCl) by annealing under N_2_ atmosphere at 600 °C for 8 h. K-PHI was synthesized from 1.21 g of 5-aminotetrazol (5-Amt), 2.25 g of LiCl, and 2.75 g of potassium chloride (KCl), which were mixed and annealed under a N_2_ atmosphere at 600 °C for 4 h. The heterojunction between K-PHI and PTI Li:Cl (denoted below as HEJ) was prepared from 0.6 g of melamine, 0.25 g of 5-aminotetrazol, 1.4 g of LiCl, and 1.7 g of KCl by annealing under a N_2_ atmosphere at 550 °C for 4 h. Prior to the annealing treatment, all precursors and salts were mixed using the ball-milling procedure (4 min, 25 Hz). All synthesized materials were exhaustively washed with deionized water and dried at 60 °C under vacuum. A more detailed description of the synthesis procedure is given in Szalad et al.^9^

## Methods

For scanning/transmission electron microscopy observations, a suspension of the sample in ethanol was sonicated for 10 min, then drop-cast onto a Cu or Au 400 mm mesh grid with a lacey carbon support film and dried for 15 min. Experiments were performed using a double Cs-corrected JEOL JEM-ARM200F (S)­TEM equipped with a cold-field emission gun, which was operated at 80 kV. Based on our calculations, presented in (Supporting Information SI), at this voltage the energy transfer from the incident beam to the target atoms is lower than at 200 kV. All calculations^36,37^ of energy transfer, ionization, and knock-on cross sections are presented in SI. Both high-resolution transmission electron microscopy (HR-TEM) images and selected area electron diffraction (SAED) patterns were recorded under low-dose conditions using a Gatan OneView camera. Details of dose rate calculations for HR-TEM imaging and SAED patterns are given in the (Table 2SI). SAED patterns were recorded with a camera length of 40 cm using Au nanoparticles (ca. 5 nm-sized Au particles sputtered onto the carbon grid) as an external standard. The Au nanoparticle standard was used for calibration at the beginning of each microscopy session following the procedure described in ref 38. A selected area aperture of 50 μm and a total exposure time of 30 s were used to record SAED patterns. They were azimuthally averaged, and the obtained scattered intensity profiles were further processed (background fitting and Fourier transformation) to get electron pair distribution functions (ePDFs) using the eRDF Analyzer software.^39^ We used the same fitting parameters for all three samples. The ePDF describes the probability of finding a pair of atoms at a particular distance, thus allowing to probe short-range order in the structure, and is especially useful for describing amorphous, poorly crystalline, and nanosized materials.^40^ The cutoff distance of the ePDF provides information about the size of coherently scattering units.

Scanning transmission electron microscopy (STEM) images were recorded using simultaneous JEOL annular dark field (ADF), bright field (BF), and secondary electron (SE) detectors. ADF-STEM images were collected with a probe convergence semiangle of 25 mrad and between 50 and 180 mrad collection semiangle.

Electron energy loss spectroscopy (EELS) data were collected using a TFS Themis Z (3.1) 80–300 microscope operated at 80 kV in monochromator mode optimized to achieve an energy resolution of ∼70 meV. The microscope is equipped with a Gatan Imaging Filter (GIF) Continuum 1065ER. The specimens were kept at a liquid nitrogen temperature of −170 °C using a Gatan Elsa 698 cryo-transfer holder. EELS spectra were recorded in dual EELS mode with energy dispersions of 0.15 and 0.3 eV/ch, allowing correction for the zero-loss peak position. A power-law model was used for background subtraction. Multiple scattering effects have been removed using the Fourier ratio method implemented in the Gatan Digital Micrograph software suite. The obtained EELS spectra were further used for the analysis of the near-edge fine structure (ELNES) and the extended electron energy loss fine structure (EXELFS). Compared to ePDF, the radial distribution functions (RDFs) derived from the EXELFS probe the short-range order with chemical specificity, thus allowing the calculation of atomic distances and coordination around chemical elements of choice. EELS data normalization for EXELFS analysis was done using the Athena software package.^41^ Extraction of the normalized cross-section χ­(*E*) was achieved by fitting two low-order polynomials to the pre- and postedge region. Normalization was done with respect to the energy-loss intensity of an isolated atom μ_0_ (in the absence of backscattering from neighboring atoms). The detected inelastically scattered electrons are described by a wavenumber *k* and a wave function χ­(*k*) that are given by the following equations:

1 $$\chi \left(E\right)=\frac{\mu \left(E\right)-\mu_{0}\left(E\right)}{\mu_{0}\left(E\right)}$$

2 $$k=\frac{2\pi }{\lambda }=\sqrt{\frac{2m}{\hbar^{2}}\left(E-E_{0}\right)}$$

where λ is the wavelength of the ejected electron, *ℏ* is the reduced Planck’s constant, *E* is the energy transfer, and *E*_0_ is the onset energy.

$$\chi \left(k\right)=\underset{j}{\sum }\frac{N_{j}{\;f}_{j}\left(k\right)}{r_{j}^{2}k}\exp\left(-\frac{2r_{j}}{\lambda_{i}}\right)\exp\left(-2\sigma_{j}^{2}k^{2}\right)\sin\left[2kr+\varphi \left(k\right)\right]$$ 3

where:

χ­(*k*) represents the EXELFS signal as a function of the electron wave vector *k*; *j* represents different neighboring atoms in the material, and the summation is taken over all neighboring atoms;

*N*_*j*_ represents the number of atoms;

$\frac{f_{j}\left(k\right)}{r_{j}^{2}k}$ represents the scattering amplitude of the *j*th atom, which depends on *k* (the wave vector) and the distance *r_j_* between the absorbing atom and the *j*th neighboring atom;

$\exp\left(-2\sigma_{j}^{2}k^{2}\right)$ is the Fourier transform of a radial broadening function that represents broadening of the RDF due to thermal, zero-point, and static disorder;

sin­[2*kr* + *φ*(*k*)] determines the interference condition.

Fourier Transforms (FT) of χ­(*k*) curves obtained from nitrogen K-edge spectra were performed by using the following normalization parameters:

*E*_0_ = 399.12 eV, *R*_bkg_ = 0.75, FT *k*-range: 1.5–8.5 Å, and *k*-weight = 2 for HEJ.

*E*_0_ = 399.5 eV, *R*_bkg_ = 0.75, FT *k*-range: 2.0–9.0 Å, and *k*-weight = 2 for K-PHI.

Theoretical EELS spectra were simulated using the *ab initio* FEFF10 code from the Demeter software suite^41^ and calculated RDFs were derived using the same workflow as for experimental EELS spectra. For these simulations, crystallographic information files (CIFs) of PTI^12,13^ and K-PHI^10^ were used as starting models. For EELS theoretical spectra calculations, the incident electron beam energy was set at 80 keV with convergence (α) and collection (β) semiangles of 22 mrad. Please note that usually RDFs refer to suitable fitting procedures of the Fourier transform of the experimental data. Because the Artemis fitting software from the Demeter project is not compatible with FEFF10 simulations, an EXELFS fitting was not performed. For simplicity, we refer to the Fourier transforms of EXELFS data as RDFs throughout the text.

High-resolution synchrotron X-ray diffraction and total scattering measurements were performed at beamline ID31 at the European Synchrotron Radiation Facility (ESRF). The sample powders were loaded into cylindrical slots (approximately 1 mm thickness) held between Kapton windows in a high-throughput sample holder. Each sample was measured in transmission with an incident X-ray energy of 75.00 keV (λ = 0.1653 Å). Measured intensities were collected using a Pilatus CdTe 2M detector (1679 × 1475 pixels, 172 × 172 μm^2^ each) positioned with the incident beam in the corner of the detector. The sample-to-detector distance was approximately 1.5 m for the high-resolution measurements and 0.3 m for the total scattering measurement. Background measurements for the empty windows were measured and subtracted. NIST SRM 660b (LaB_6_) was used for geometry calibration, performed with the software pyFAI, followed by image integration including flat-field, geometry, solid-angle, and polarization corrections.

## Supplementary Material



## Notes

Detailed supplementary results, including 9 figures, 4 tables, a detailed description of acquisition parameters, and the estimation of knock-on and ionization cross sections at different voltages (PDF)
