# Supplementary material for: Site Defects and Structural Alignment Enhance Interfacial Charge Mobility in Heterostructured Carbon Nitride Catalysts
Source: ACS Nano. 2026 Jan 5;20(2):2125–36. doi: 10.1021/acsnano.5c15285 (PMC12825372; doi:10.1021/acsnano.5c15285)
Supplement: Supplementary file 1 [file nn5c15285_si_001.pdf]

Supporting Information

Site defects and structural alignment enhance interfacial charge mobility in heterostructured carbon nitrides catalyst

Teodor Jianu[1,*], *Horațiu Szalad*[1], *Vladimir Roddati*[4], *Markus Antonietti*[1], *Nadezda V. Tarakina*[1,2,3*]

[1]Department of Colloid Chemistry, Max Planck Institute of Colloids and Interfaces, 14476 Potsdam, Germany

[2]INM-Leibniz Institute for New Materials, Campus D2 2, 66123 Saarbrücken, Germany

[3]Department of Materials Science and Engineering, Saarland University, 66123 Saarbrücken, Germany

[4]GFZ Helmholtz Centre for Geosciences, Telegrafenberg, 14473 Potsdam, Germany

* - To whom correspondence should be addressed: teodor.jianu@mpikg.mpg.de and nadja.tarakina@leibniz-inm.de

Dose rate calculation

For SAED patterns and TEM images, the dose rate, expressed as $e^{-}/Å^{2}/s$ is calculated as follows:

A TEM 60kX image with the Condenser Lens number 3 (CL3) value set to corresponding focus conditions in diffraction mode is recorded for 1s. The illumination area covers the whole One View camera, ensuring that all the beam interacting with the specimen is measured. Using GMS 3.0 option of electron counting in a specified area, a mean value of e^{-}/pixel is obtained. Then the pixel size is converted to $Å^{2}$ and the required division is done. During acquisition of TEM images or SAED patterns the Cl3 values was not changed.

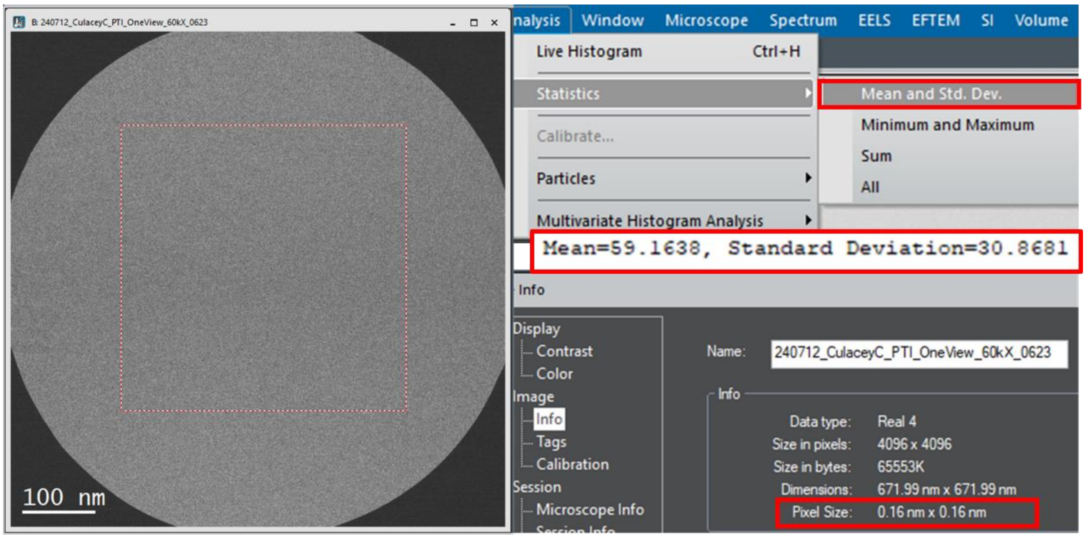

Figure 1SI. Example of mean electron count/pixel and corresponding size,

Table 1 SI. Dose rate for HR and overview TEM images and SAED patterns calculated using the acquisition parameters for measurements used throughout the study.

Sample	HR-TEM dose rate $[e^{-}/Å^{2}/s]$	Overview TEM dose rate $[e^{-}/Å^{2}/s]$	Diffraction dose rate $[e^{-}/Å^{2}/s]$
K-PHI	3	10	22.5
HEJ	2	11	23.1
PTI	2	3	20.9

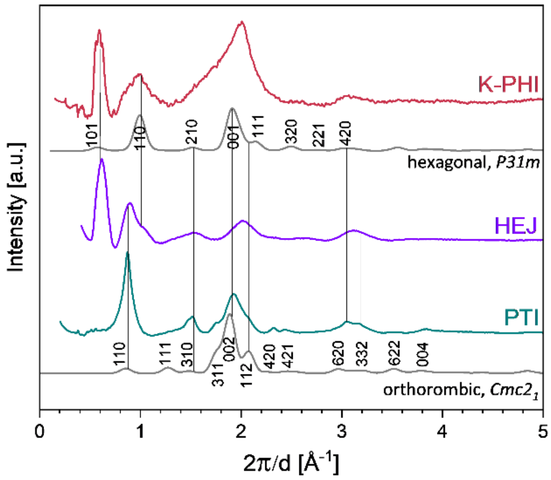

Figure 2SI. Azimuthally averaged SAED patterns of K-PHI (red), PTI (cyan) and heterojunction (purple). Diffraction peaks of the phases are assigned based on the theoretical diffraction profiles (grey).

K-PHI model: Angew.Chem. Int. Ed. **2020**, 59,15061–15068 (ref. 9 in the main text)
PTI model: Inorg. Chem. 2019, 58, 15880−15888 (ref. 12 in the main text)

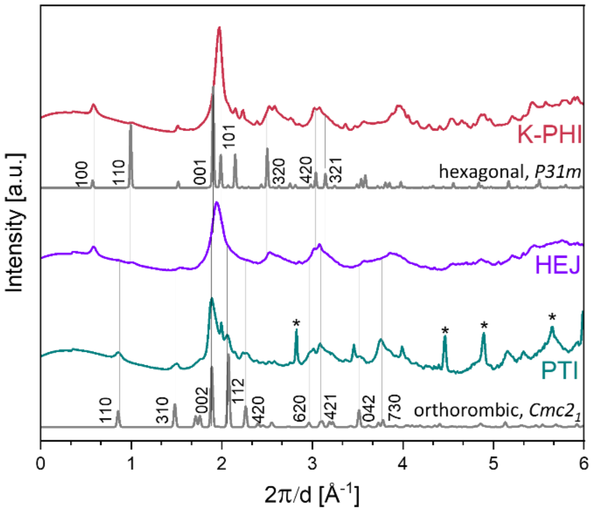

Figure 3SI. Synchrotron-based X-ray scattering profiles of K-PHI (red), PTI (cyan) and heterojunction (purple). Diffraction peaks of the phases are assigned based on the theoretical diffraction profiles (grey).
The * on top of sharp diffraction peaks correspond to projections from LiCl crystallographic planes. The salt was not removed completely during the washing stage of the synthetic procedure.

For EELS dose calculation, the pixel size is given in nm^2 and the electron count is recorded for each pixel in the Spectroscopy Image (SI). The dose varies with the exposure in low-loss or core-loss regime.

Table 2 SI. Dose calculation for EELS spectra used throughout the study.

Sample	K-PHI	HEJ	PTI
pixel size [nm^2]	15	19	25
Low Loss exposure [s]	0.0005		
e^- counts / pixel	16.8	15.7	14.5
Dose [e^-\nm^2]	1.1	0.8	0.6
Core Loss exposure [s]	0.01	0.02	0.05
e^- counts / pixel	7.3	7.0	9.7
Dose [e^-\nm^2]	0.5	0.4	0.4

Table 3SI. Average width of aloof regions used for π-plasmons VEELS analysis.

Width aloof region			
Sample	K-PHI	HEJ	PTI
Min. dist. from particle [nm]	11.1	9.8	8.5
Max. dist. from particle [nm]	27.3	26.1	26.7
Width = Max – Min [nm]	16.2	16.3	18.1
Thickness [t/λ]	0.01	0.01	0.01

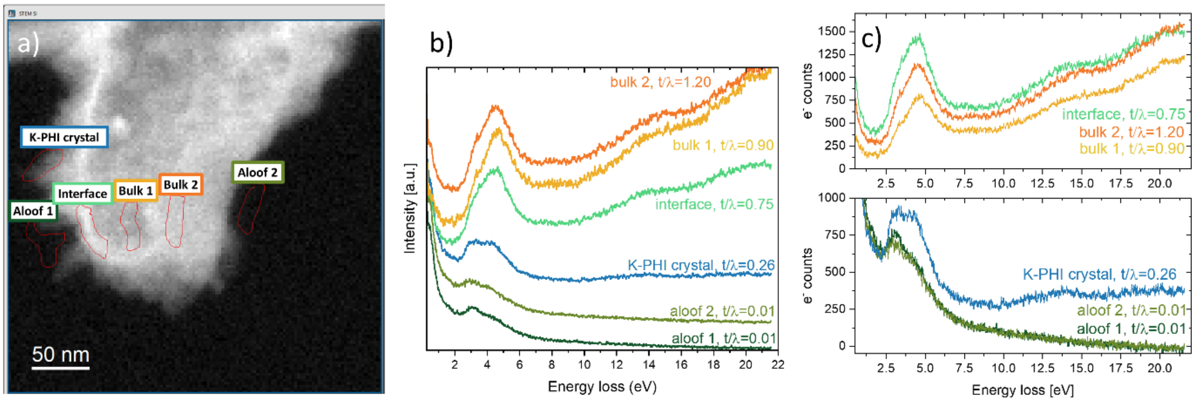

Figure 4SI. (a) regions of the particle with different thicknesses (expressed as t/λ); (b) corresponding normalized VEELS spectra showing the plasmons responses and thickness values; (c) as-recorded EELS spectra showing the most intense π-plasmons response from the interface region of the particle.

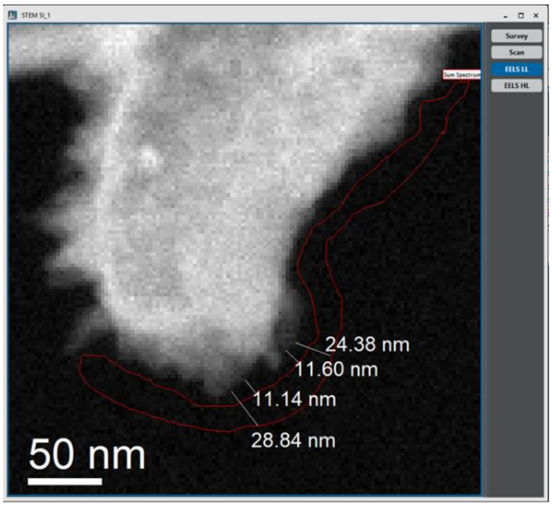

Figure 5SI. Example of region around heterojunction particle for aloof-VEELS spectra analysis.

Table 4SI. ELNES values for all three samples at the C and N K-edges and assigned chemical bonding.

	K-PHI	*Bonding*	HEJ	*Bonding*	PTI	*Bonding*
**C edge**	286,2	CHx	286,2	CHx	286,8	C=O/C-O
	287,0	N-C=C	287,0	N-C=C	-	-
	288,0	C-NHx	288,1	C-NHx	288,1	C-NHx
**N edge**	400,4	C-N=C	400,4	C-N=C	400,4	C-N=C
	401,8	N-Hx	401,8	N-Hx	401,8	N-Hx
	402,6	N quaternary	401,4	N quaternary	-	-

a)

N K-edge	K-PHI	H-025	PTI
I(sp2)	220.9	160.8	75.6
I(σ)	178.6	150.5	100.9
Ratios	1.23	1.06	0.74

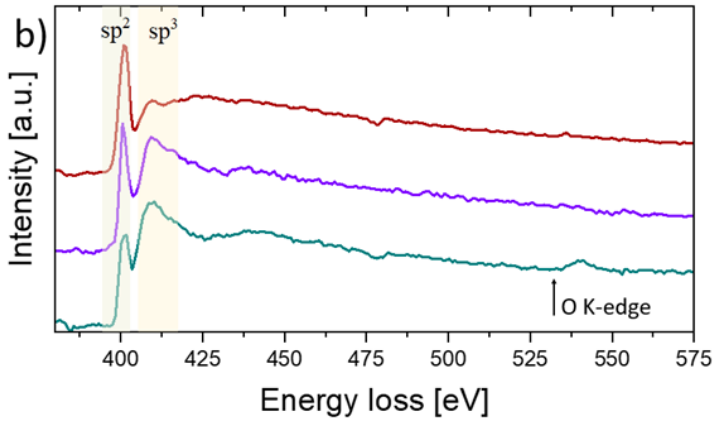

Figure 6SI (a) this table summarizes intensities values of sp^2 and sp^3 contributions derived from the N K-edge EELS spectra shown in (b).

PTI ELNES mapping

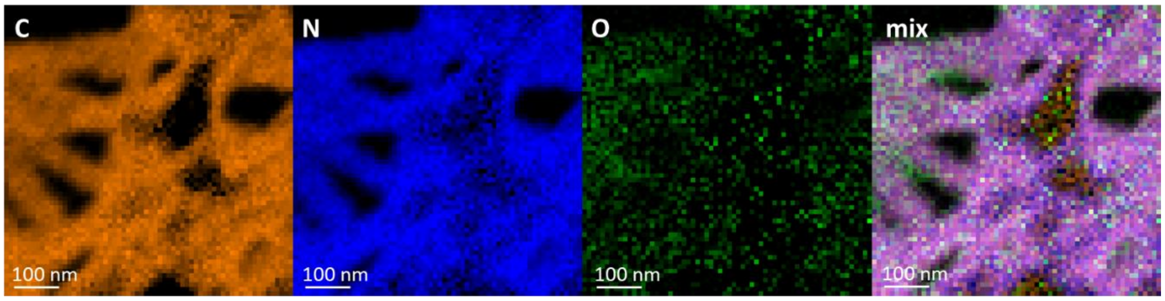

Figure 7SI. ELNES mapping of C, N and O K-edges in the PTI sample, showing the presence of O at the surface of particles.

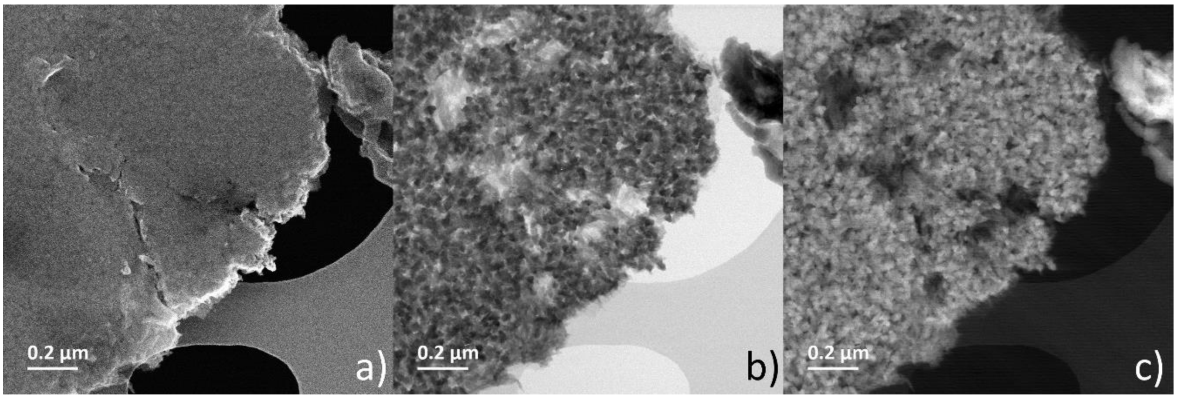

Figure 7SI. a) Secondary electrons image showing the flat surface of heterojunction made of PTI layer and K-PHI crystallites growing inside visible in b) BF and c) ADF images.

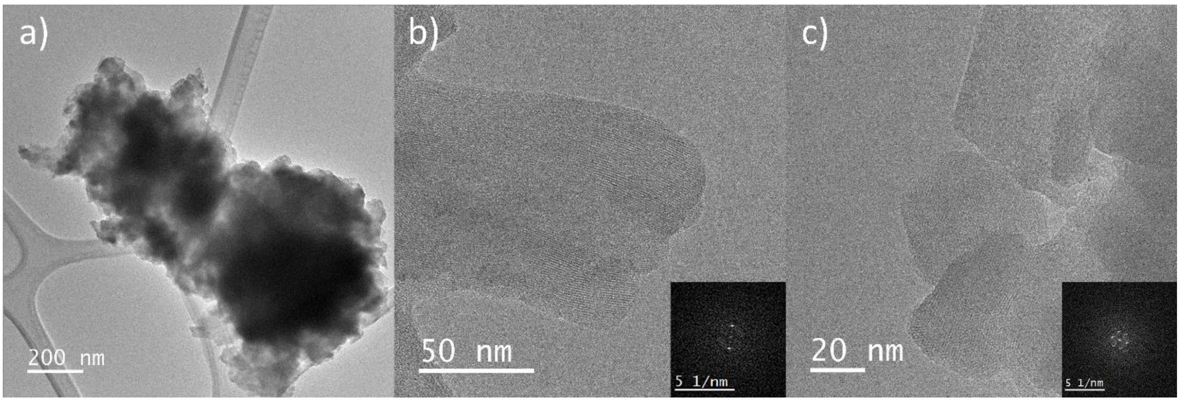

Figure 8SI. a) K-PHI overview TEM image showing a particle made of agglomerated crystallites; b) and c) HR-TEM images and FFTs displaying two different orientations of the crystals.

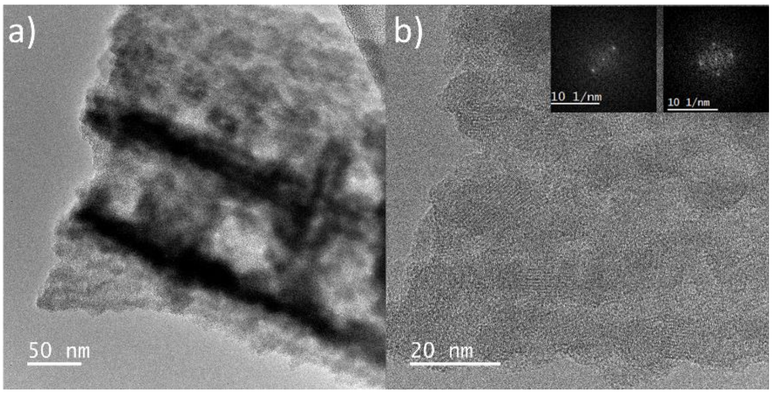

Figure 9SI. a) PTI overview TEM image and b) HR-TEM images and FFTs displaying two different orientations of the crystals.

The calculations listed below were carried out using E-beam Calculator[1].The calculator is based on earlier published works by McKinley et al.[2],and Kim et al[3].

Ionization cross-section

Evaluation of the ionization probability of atomic orbitals induced by electron beams was done using the relativistic Binary Encounter Bethe (σ_{rBEB}) model proposed by Kim et al[3]:

$$\sigma_{rBEB} = \frac{4\pi a_0^2 \alpha^4 N}{(\beta_t^2 + \beta_u^2 + \beta_b^2)\, 2b'} \left\{ \frac{1}{2}\left[ln\left(\frac{\beta_t^2}{1-\beta_t^2}\right) - \beta_t^2 - ln(2b') \right]\left(1 - \frac{1}{t^2}\right) + 1 - \frac{1}{t} - \frac{ln\, t}{t+1} \frac{1+2t'}{\left(1+\frac{t'}{2}\right)^2} + \frac{b'^2}{\left(1+\frac{t'}{2}\right)^2} \frac{t-1}{2} \right\} \quad \text{Eq. S1}$$

where a_0 is the Bohr radius = 0.5292 Å, N is the electron occupation number, E_e is the indecent electron energy, B is the orbital binding energy and U is the orbital kinetic energy.

The factors used in the equation are defined as:

$$\beta_t^2 = 1 - \frac{1}{(1+t')^2} \qquad \beta_b^2 = 1 - \frac{1}{(1+b')^2} \qquad \beta_u^2 = 1 - \frac{1}{(1+u')^2}$$

$$t' = \frac{Ee}{mc^2} \qquad b' = \frac{B}{mc^2} \qquad u' = \frac{U}{mc^2}$$

Orbital binding energy (B) of VB/HOMO level = 1.73eV [4]

Orbital kinetic energy (U) = (Z_{eff}^2 x 13.6)/n^2

Here, Z_{eff} is the effective nuclear charge accounting for screening from the other electrons, it can be estimated using Slater's rule[5].

**Atom**	**Orbital**	**n**	**Z_{eff}**	**U(eV)**	**σ_{rBEB} (@200kV)**	**σ_{rBEB} (@80kV)**
C	2p	2	3.25	35.9	4.07e-2 Å^2	7.33e-2 Å^2
N	2p	2	3.9	51.7	4.07e-2 Å^2	7.33e-2 Å^2
K	$4s^1$	4	2.2	4.11	4.07e-2 Å^2	7.34e-2 Å^2

Orbital binding energy (B) of 1s core-level: 285 eV for C, 399.3 eV for N

Orbital binding energy (B) of 2p core-level: 294 eV for K

**Atom**	**Orbital**	**n**	**Z_{eff}**	**U (eV)**	**σ_{rBEB} (@200kV)**	**σ_{rBEB} (@80kV**
C	1s	1	6	489.6	1.51e-4 Å^2	2.59e-4Å^2
N	1s	1	7	666.4	1.03e-4 Å^2	1.76e-4 Å^2
K	2p	2	14.85	749.7	1.46e-4 Å^2	2.49e-4 Å^2

Knock-on cross section

The energy-momentum conservation relation (Eq. S2) in elastic scattering events can be used to estimate the kinetic energy transferred from the incident electron to the target atoms[2]:

$$E(E_e, \theta, \nu) = \frac{2[E_e\,(E_e + 2m_0c^2) + \sqrt{E_e + 2m_0c^2 M\nu c}](1-\cos\theta) + (M\nu c)^2}{2Mc^2} \qquad \text{Eq. S2}$$

Where E_e is the incident electron energy, m_0 is the electron rest mass = 0.511 x 10^6 [eV/c^2], c is the speed of light = 2.998 x 10^8 [m/s], θ is the scattering angle, ν is the velocity of the target atom and M is the mass of the target atom.

The maximum energy transfer occurs in backscattering events when θ = 180°. The maximum energy can be calculated and the equation can be simplified to:

$$E_{max}(E_e, \theta = 180^\circ, \nu) = \frac{\left(2\sqrt{E_e(E_e+2m_0c^2)} + M\nu c\right)^2}{Mc^2} \qquad \text{Eq. S3}$$

We can assume that the speed of the target nucleus is significantly slower than that of the incident electron, so ν = 0. The maximum energy transfer can be calculated using Eq. S4:

$$E_{max}\,(E_e, \theta = 180^\circ, \nu=0) = \frac{2E_e\left(E_e+2m_0c^2\right)}{Mc^2} \qquad \text{Eq. S4}$$

If the energy transfers from the electron beam exceeds the bond threshold displacement energy (E_d), a knock-on damage can occur. The knock-on cross section (σ_{KO}) can be calculated using Eq. S5

$$\sigma_{KO}(E_e, \nu) = 4\pi\left(\frac{Ze^2}{4\pi\varepsilon_0 2\gamma m_0 c^2\beta^2}\right)^2\left[\left(\frac{E_{max}}{E_{KO}} - 1\right) - \beta^2 ln\left(\frac{E_{max}}{E_{KO}}\right) + \pi Z\alpha\beta\left\{2\left(\sqrt{\frac{E_{max}}{E_{KO}}} - 1\right) ln\left(\frac{E_{max}}{E_{KO}}\right)\right\}\right] \qquad \text{Eq. S5}$$

Where Z is the atomic number, ε0 is the vacuum permittivity = 55.263 x 10^6[eV/c^2], $\gamma = \frac{1}{\sqrt{1-\beta^2}}$ is the Lorentz factor with $\beta = \sqrt{1-(1+\frac{E_e}{m_0c^2})}$ the relativistic factor and $\alpha = \frac{e^2}{4\pi\varepsilon_0\hbar c}$ = 0.007297352 is the fine structure constant.

As there are no computed bond energy values for carbon nitride materials, we estimate the energy transfer and knock-on cross sections using typical C-N and C=N bond energies.

C-N = 305kJ/mol=5.23eV/atom

C=N = 615kJ/mol=6.37eV/atom

Atom	E_d [eV/atom]	σ_{KO} (80kV) [Å^2]	Emax (80kV) [eV]	σ_{KO} (200kV) [Å^2]	Emax (200kV) [eV]
C	C-N = 5.23<br>C=N = 6.37	1.86e-6<br>1.33e-6	15.77	1.29e-6<br>1.00e-6	43.73

N	C-N = 5.23<br>C=N = 6.37	1.96e-6<br>1.36e-6	13.52	1.45e-6<br>1.12e-6	37.48

For example, energy transfer at θ = 10° (much higher than usual elastic scattering events)

80kV: C = 0.12eV, N = 0.10eV

200kV: C = 0.33eV, N = 0.28eV

An incident beam at 80kV and scattering angle θ = 10° has an energy transfer value of ~0.11eV for light elements compared to ~0.3eV at 200kV, thus much lower than E_d for C-N and C=N bonds. At 80kV the ionization cross-section of the HOMO or VB levels is 4 orders of magnitude larger than the knock-on cross section, clearly proving that an ionization process is ~10000 times more probable to occur than a knock-on displacement during an electron-target atom interaction.

REFERENCES

1. Kim, Y. K., Santos, J. P. & Parente, F. Extension of the binary-encounter-dipole model to relativistic incident electrons. *Phys. Rev. A - At. Mol. Opt. Phys.* **62**, 052710–052711 (2000).
2. Szalad, H. *et al.* Polymeric triazine/heptazine imide heterostructures enable photocatalytic O2 reduction to H2O2. *Appl. Catal. B Environ.* **357**, (2024).
3. Slater, J. C. Atomic shielding constants. *Phys. Rev.* **36**, 57–64 (1930).
4. William A. McKinley Jr., H. F. The Coulomb Scattering of Relativistic Electrons by Nuclei. *Phys. Rev.* **74**, 1759–1763 (1948).
